# Public perception and acceptance of coypu *Myocastor coypus* removal in urban areas: influences of age and education

**DOI:** 10.1007/s00114-024-01928-2

**Published:** 2024-08-02

**Authors:** Andrea Viviano, Isabella De Meo, Emiliano Mori, Carlotta Sergiacomi, Alessandro Paletto

**Affiliations:** 1 Consiglio Nazionale delle Ricerche, Istituto di Ricerca sugli Ecosistemi Terrestri, Sesto Fiorentino (Firenze), Italy; 2 Research Centre for Agriculture and Environment, Consiglio per la Ricerca in Agricoltura e l’Analisi dell’Economia Agraria (CREA), Firenze, Italy; 3 National Biodiversity Future Center (NBFC), Palermo, Italy; 4 Research Centre for Forestry and Wood, Consiglio per la Ricerca in Agricoltura e l’Analisi dell’Economia Agraria (CREA), Trento, Italy

**Keywords:** Alien species, Urban biodiversity, Coypu management, Questionnaire survey, Tuscany region (Italy)

## Abstract

Monitoring and management of alien coypu (*Myocastor coypus*) is a key issue in Europe since this species has been included in the EU Invasive Alien Species Regulation 1143/2014. Thus, controlling the population of this rodent is considered as imperative by wildlife managers. Coypu management in urban areas is crucial considering potential conflicts with human activities. The aim of this study is to investigate citizens’ knowledge, perceptions and opinions towards the presence and management of coypu in a case study in Central Italy (the Serravalle urban park, Tuscany). The survey was administered to a subset of municipal residents and garnered responses from 281 park visitors. The outcomes showed a high level of knowledge of respondents: 99.3% of total respondents had heard of coypu before this survey, and 93.9% were able to distinguish the coypu from the Eurasian beaver (*Castor fiber*). Despite the importance of coypu control in invaded countries, our results highlight that most respondents are against the removal of coypu, with the exception of most young, highly-educated and upperclass men, but favouring sterilization over capture and population control. Findings of this study could assist managers in enhancing the efficacy of management efforts via an information initiative involving the general public. Achieving this goal may entail improved communication by wildlife managers, which is imperative for optimizing management strategies regarding alien species.

**Supplementary information:**

The online version contains supplementary material available at 10.1007/s00114-024-01928-2.

## Introduction

Biological invasions are considered one of the five most important direct drivers of biodiversity loss as-well-as habitat change, climate change, overexploitation, and pollution (Katsanevakis et al. 2013; Henry et al. 2023). In Europe, over 10,000 plant and animal alien species that have a negative impact on human activities and the environment have been estimated to occur, of which approximately 15% have become invasive (Boase 2015). The European Union (EU) Biodiversity Strategy 2020 emphasized the need to assess pathways of biological invasions, as required by the Target 5 (EC 2011). Afterwards, the EU Invasive Alien Species Regulation (Regulation 1143/2014) focused on the prevention and management of the introduction and spread of invasive alien species.

The coypu (*Myocastor coypus*) is a large-sized rodent – which can reach 4–5 kg in adult free-ranging individuals and up to 12 kg in captivity (Prigioni et al. 2005a; Amori et al. 2008) – whose native range includes several countries of South America (i.e., Southern Brazil, Chile, Bolivia, Paraguay, Argentina, and Uruguay). During the XVIII and XIX centuries, the coypu has been introduced throughout Eurasia, North America, and part of Africa for fur farming (Carter and Leonard 2002; Schertler et al. 2020; Pedruzzi et al. 2022). Coypus are semi-aquatic rodents which spend most of their time foraging along rivers, ponds, and lakes, from rural to suburban and urban areas, showing a wide ecological plasticity (D’Adamo et al. 2000; Mori et al. 2020; Poláčková et al. 2022; Salas et al. 2022). Where released by humans, coypus may affect native biodiversity and environments by: feeding on local vegetation (Prigioni et al. 2005b) and cultivated species (D’Adamo et al. 2000; Panzacchi et al. 2007); crashing the eggs of waterbirds by trampling them on bird floating nests (Angelici et al. 2012; Bertolino et al. 2012); transmitting diseases to other species and zoonoses (e.g., leptospirosis: Vein et al. 2014); burrowing in riverbanks and railway stations, threatening their integrity (Carter and Leonard 2002; Dondina et al. 2024); inducing divergence in the natural equilibrium between autochthonous prey and predators, resulting in reduced predation pressure on native species (Ferretti et al. 2019; Mori et al. 2022). The coypu was therefore included in the list of invasive alien species established by the Regulation 1143/2014 in 2016.

Several studies occur in the scientific literature on the health status of coypu (Bollo et al. 2003; Martino et al. 2014; Nicoletti et al. 2024) and on the distribution and management of alien populations (Carter and Leonard 2002; Corriale et al. 2006; Schertler et al. 2020; Pedruzzi et al. 2022). Bertolino and Viterbi (2010) showed a cost-benefit analysis of an effective permanent control program of coypu to limit crop damages in northern Italy. Afterwards, Walther et al. (2011) analysed the measures taken to eradicate coypu from urban environments, primarily focusing on the prohibition for citizens to feed them.

Successful management often relies on community involvement and a shared understanding of the ecological and economic threats posed by these invaders (La Morgia et al. 2017; Cerri et al. 2020; Rodríguez-Rey et al. 2022). Thus, assessing public perception of invasive alien species, mostly in terms of acceptance of removal and control strategies, is crucial for effective management (Bennett 2016), as people views on these species may influence their willingness to participate in control programs, report sightings, or even support eradication efforts (Novoa et al. 2017; Rodríguez-Rey et al. 2022; Haley et al. 2023). Understanding these perceptions can help tailor communication strategies to address concerns and build public trust in the importance of managing invasive species (Estévez et al. 2015; Kapitza et al. 2019). Besides a general agreement amongst researchers (Cerri et al. 2024), thanks to well-designed communication campaigns, public awareness of the problems caused by biological invasions has grown significantly in recent times, particularly regarding young, more educated individuals and/or upperclassmen (Sharp et al. 2011; Waliczek et al. 2017; Lioy et al. 2019).

While some studies were conducted on the social perception of other semiaquatic rodent species (Garba et al. 2014; Capobianco et al. 2023), very few studies concern the perception of coypu (Adriani et al. 2011; Liordos et al. 2017). Some attention is given to the perception of different management strategies, by using face-to-face interviews to gauge public opinion on these methods (Liordos et al. 2017; Ulicsni et al. 2020; Viviano et al. 2023). Urban areas are particularly suitable for testing human perception of invasive alien species, as they host the vast majority of local escapes or releases (Padayachee et al. 2017; Potgieter et al. 2019). Coypus demonstrate significant adaptability to urban environments, both in their native habitats and non-native territories (Meyer et al. 2005; Corriale et al. 2006), leading to frequent interactions with humans (Walther et al. 2011; Salas et al. 2022) who often feed them (Meyer et al. 2005; Walther et al. 2011). Particularly in Italy the species is recorded in a high number of cities (e.g., Roma, Milano, Torino, Firenze, Bologna, Verona, Pisa, and Grosseto, and several towns: Burchielli 2023). In all these cities information on public perception of coypu management is lacking while coypus are abundant in urban parks, near humans engaged in various activities (Burchielli 2023). Given the potential for negative impacts, evaluating public perception of the coypu as an invasive alien species is crucial to inform on the feasibility and efficacy of control strategies, which are imperative wherever practicable. This is particularly relevant in urban environments, where the risk of zoonotic disease transmission and detrimental effects on human well-being are inherently amplified. Thus, aim of our study was to delve into the public perception of coypus within an urban setting, contributing to an area of research that has not yet been extensively explored in current literature. To this end, the research questions were: (R1) Does citizens’ level of knowledge about the coypu influence their perception of this species? (R2) Do human emotions towards coypu influence management preferences of this species at the local level? To achieve this aim, a questionnaire survey was conducted among a sample of citizens in a case study in central Italy, specifically in the Serravalle urban park in the Tuscany region. This park is characterized by significant human activity and a notable population density of coypus, rendering it an ideal location to examine social attitudes toward this invasive rodent. Given the recent increase in communication about alien species (e.g., Piria et al. 2017; De Groot et al. 2020), we predicted that young and highly educated individuals would be the most aware of the problem and therefore more inclined to accept alien species removal actions.

## Materials and methods

### Study area

The survey was conducted in Tuscany, in the Serravalle urban park in Empoli municipality (23 ha, 43.728°N-10.964°E, 36 m asl), given its local high density of coypus and the high number of annual visitors and tourists. The Park includes an artificial pond of about 1.2 hectares in its southernmost part, surrounded by a meadow with *Cerastium glomeratum* Thuill., *Trifolium repens* L., *Trifolium nigrescens* Viv., *Trifolium pratense* L., *Leontodon tuberosus* L., *Plantago coronopus* L. and *Erodium malacoides* (L.) L'Hér (Peruzzi 2023). The pond was once linked to another wetland (Arnovecchio), located about 2 km eastwards, which supported the arrival of the coypu to the Serravalle urban park. In the former area, the high-water depth (over 3 m) due to excavation activity did not support anymore the presence of coypus. Thus, the local coypu population may represent an isolated nucleus of this species, with poor connections with other populations.

In the northernmost part of the study area and around the lake there is a wooded area covering about 30% of the park with several tree and shrub species, including *Pinus pinea* L.*, Quercus ilex* L.*, Quercus pubescens* Willd.*, Populus nigra* L.*, Arbutus unedo* L. and *Punica granatum* L. To the east of the lake, a small cultivation (1.5 ha) of maize (*Zea mays* L.) is consumed by coypus in summer.

Within the park, at least 10–15 coypus occur, together with 25–30 mallards (*Anas platyrhynchos*), four Muscovy ducks (*Cairina moschata*), a shelduck (*Tadorna tadorna*), a swan goose (*Anser cygnoides*), four domestic geese (*Anser anser*), and terrapins of the alien species *Trachemys scripta*. Other wetland birds occurring in the lake include the grey heron (*Ardea cinerea*), the egret (*Egretta garzetta*), the black-headed gull (*Chroicocephalus ridibundus*), the Mediterranean yellow-legged gull (*Larus michahellis*), the common sandpiper (*Actitis hypoleucos*), and the kingfisher (*Alcedo atthis*). Along the shores of the pond, at least 29 coypu den entrances were counted in 2023, all of which were used. Other mammals include the Eastern cottontail (*Sylvilagus floridanus*), the red fox (*Vulpes vulpes*) and some unleashed domestic dogs (*Canis familiaris*) (Burchielli 2023).

The Serravalle urban park is the main city reference point for outdoor sports and recreational activities in Empoli. Therefore, the human presence is abundant, with access by up to 3,000 people per day in summer. As a result, wildlife is often fed by humans with fruit or vegetable scraps and bread and this may help a future further growth of the local coypu population (Guichón et al. 2003). Furthermore, this can lead to direct interactions between people and animals (including coypus), which come closer in search of food.

### Questionnaire survey

The present survey was implemented in September-December 2023 in the framework of the National Recovery and Resilience Plan (PNRR)-National Biodiversity Future Centre, which aims at investigating different management actions for the fauna of sub-urban areas with high anthropization. The survey was addressed to visitors of the Serravalle urban park to investigate public knowledge, perceptions and opinions towards the presence and management of coypu in the park. The design of the survey was made by a working group, composed by researchers from the Italian Council for Agricultural Research and Economics (CREA), the Italian National Research Council (CNR, Institute of Research on Terrestrial Ecosystems), and the National Biodiversity Future Centre. The working group opted to employ an online survey tool, utilizing a questionnaire format, due to the inherent advantages of this quantitative method. These include a direct connection to respondents who are geographically separated, the facility of access for respondents, the opportunity to reduce time and costs, and the directness of the research process and public control of ethical standard (Wright 2005; Paletto et al. 2022).

A pre‐test phase was conducted over a two-week period to verify the precision and adequacy of the questionnaire, adopting a supervised method approach (Fischl 2013). The questionnaire was pre-tested with a convenience sample of seven known respondents, who filled out the online questionnaire while the authors monitored any problems. The respondents completed the questionnaire on a tablet in the presence of a member of the working group and then they discussed difficulties or doubts. The final version of the questionnaire (Supplementary Material 1) was formed by 16 closed-ended questions to be easily understood by people of any social and educational background. The questionnaire survey can be accessed through the following link in Italian language (https://ec.europa.eu/eusurvey/runner/6f31571d-d918-9cae-9cd7-f6312b5498a7).

The questionnaire was structured in three sections and began by asking whether the interviewee had actually visited the park, influencing the rest of the survey responses (see Supplementary Material 1). The first section was formed by three questions aimed to explore the knowledge of respondents about the coypu. The second section of the questionnaire consisted of six questions aimed at investigating respondents’ perceptions and linked reasons about the presence of coypu in the study area. The emotions experienced when seeing a coypu were also explored. In this case, the respondents were asked to evaluate the level of emotions they feel when seeing a coypu using a seven-point Likert scale format (from 0 “no emotion” to 7 “very strong emotion”). The choice was among: “joy”; “fear”; “guilt”, “repugnance/disgust”; “love”; “compassion”. Emotions were chosen based on the list of primary emotions following Scarantino and Griffiths (2011), to ensure the replicability of the study, as the primary emotions are a well-established and widely accepted set of emotions. For this study, emotions were selected based on their relevance to human-wildlife interactions, as they are all emotions that are commonly experienced by humans in relation to animals (Jacobs and Vaske 2019). For example, fear is a common emotion in humans when interacting with wildlife, as it can be a dangerous encounter (Lasky and Bombaci 2023), as well as disgust towards certain species of insects (Notaro et al. 2023). Anger is another common emotion, as it can be elicited by feeling threatened or frustrated by an animal (Lute et al. 2016). Joy is also a common emotion in human-wildlife interactions, as it can be experienced when observing or interacting with animals in a positive way (Jacobs and Vaske 2019). By selecting emotions that are both well-established and relevant to human-wildlife interactions, we were able to ensure that our study was both reliable and valid. Then, respondents’ preferences towards coypu management strategies were explored. Visitors were asked to express their opinion concerning the removal of the coypu from the Serravalle urban park, estimating their level of approval of different management actions using a five-point Likert scale format (from 1 “total disapproval” to 5 “total approval”). With the third and last thematic section the personal information of respondents was collected.

### Data collection

The electronic survey was uploaded to the EUSurvey online platform (https://ec.europa.eu/eusurvey/), the official online platform for surveys provided by the European Commission. The online questionnaire was made available only to adult visitors (aged > 18 years) of the Serravalle urban park for three months, from early September to the end of December 2023. The survey was conducted in compliance with EU regulation 2016/679 of the European Parliament on the protection of individuals regarding the treatment of their personal data. No informed written consent was requested because the online survey was anonymous, and no personal data were collected. All respondents were informed of the aim of the survey, which is not of a commercial or advertising nature but purely scientific, and that the data collected would be processed and disclosed only in aggregate form, in accordance with the Italian Law 196/2003. In case during the compilation respondents change their minds, they could leave the survey at any time before clicking the “submit” button and sending their answers. In this case, the information was not saved. Each completed questionnaire was submitted to the EUSurvey online platform, and the final database was downloaded as a Microsoft Excel sheet.

The survey was administered both in person at the Serravalle Park, stopping park visitors and using a tablet, and via social media on the dedicated pages of the Municipality of Empoli. The first question (before the actual questionnaire) asked respondents if they had visited the park, and only those who had were allowed to answer subsequent questions. Direct links to the electronic survey were distributed using various online services, including public Facebook pages/groups. At the end of the data collection period, i.e., in four months, 281 park visitors filled out all questions of the questionnaire which constitute the sample of the study. Other 19 visitors left part of the survey unfilled, thus their answers were discarded from further analyses. The 281 questionnaires collected in this study are comparable with the samples used in other similar studies in urban parks or small study sites (Cerri et al. 2020; Gargioni et al. 2021; Sogliani et al. 2021).

### Data analyses

In the final stage, the collected data were processed to generate key descriptive statistics: mean, median, and standard deviation (SD) for data gathered via the Likert-scale format, and frequency distribution (%) for other inquiries. In particular, the level of the emotions towards coypu was calculated considering that the data was collected using a 7-point Likert scale – from no emotion to a very strong emotion – and the type of emotions (positive such as “joy” and “love”; negative such as “fear”, “guilt”, “disgust” and “compassion”). From the statistical point of view, two non-parametric tests were performed to highlight statistically significant differences between groups for responses collected using the Likert scale format (Questions Q2.2, Q2.3.1, Q2.3.2 in Supplementary Material 1). In particular, the non-parametric Kruskal‐Wallis test (*α* = 0.05) was used to highlight statistical significant differences considering age, level of education, and occupation; while the non-parametric Mann-Whitney test (*α* = 0.05) was used to point out statistical significant differences considering gender and city of residence. The non-parametric tests were applied, rather than parametric tests, for the following two reasons: the sample size is not large enough (281 questionnaires collected); the assumption of normality is violated considering the results of the Shapiro-Wilk test (*p* < 0.0001). In addition, the Chi-square (*χ*^*2*^) test was applied for single choice questions (Questions Q1.2, Q2.1). The linear regression model and the Spearman correlation test (*α* = 0.05) were used to analyse the relationships between level of knowledge and preferred management strategies, and emotions towards coypu. The individual level of knowledge towards the coypu was calculated as the sum of the answers to questions from Questions Q1.2a to Q1.2d by assigning 1 if the answer given was correct (“Beaver” for Q1.2a; “Both” for Q1.2b; “Beaver” for Q1.2c; and “Coypu” for Q1.2d), -1 if it was incorrect and 0 in case of “I don't know”. Therefore, the aggregate level of knowledge based on the four questions could range from -4 to + 4. All statistical tests were performed using the XLStat 2020 software.

## Results

### Socio-demographic characteristics of respondents

Throughout the data collection process, a total of 281 questionnaires were completed in their entirety and subsequently processed, with most respondents being between 25 and 34 years old, having a university or post-university degree and employed in the private or in the public sector. Most respondents are residents in the Empoli municipality (Table 1). Data were not transformed for further analyses (shown in the following paragraphs).

**Table 1:** Socio-demographic characteristics of respondents

**Gender**	**Male**	**Female**				
	36.9%	63.1%				
**Age class**	**18–25**	**25–34**	**35–44**	**45–54**	**55–64**	**> 65**
	7.1%	22.9%	22.1%	20.4%	21.1%	6.4%
**Education**	**Elementary**	**High school**	**University and post-university**			
	8.2%	44.3%	47.5%			
**Occupation**	**Unemployed**	**Retired**	**Student**	**Public sector**	**Private sector**	
	6.8%	7.5%	10.0%	24.3%	51.4%	
**City of residence**	**Empoli**	**Others in Tuscany**	**Others in Italy**			
	59.6%	40.4%	5.0%			

### Level of knowledge about coypu

**Fig. 1:**
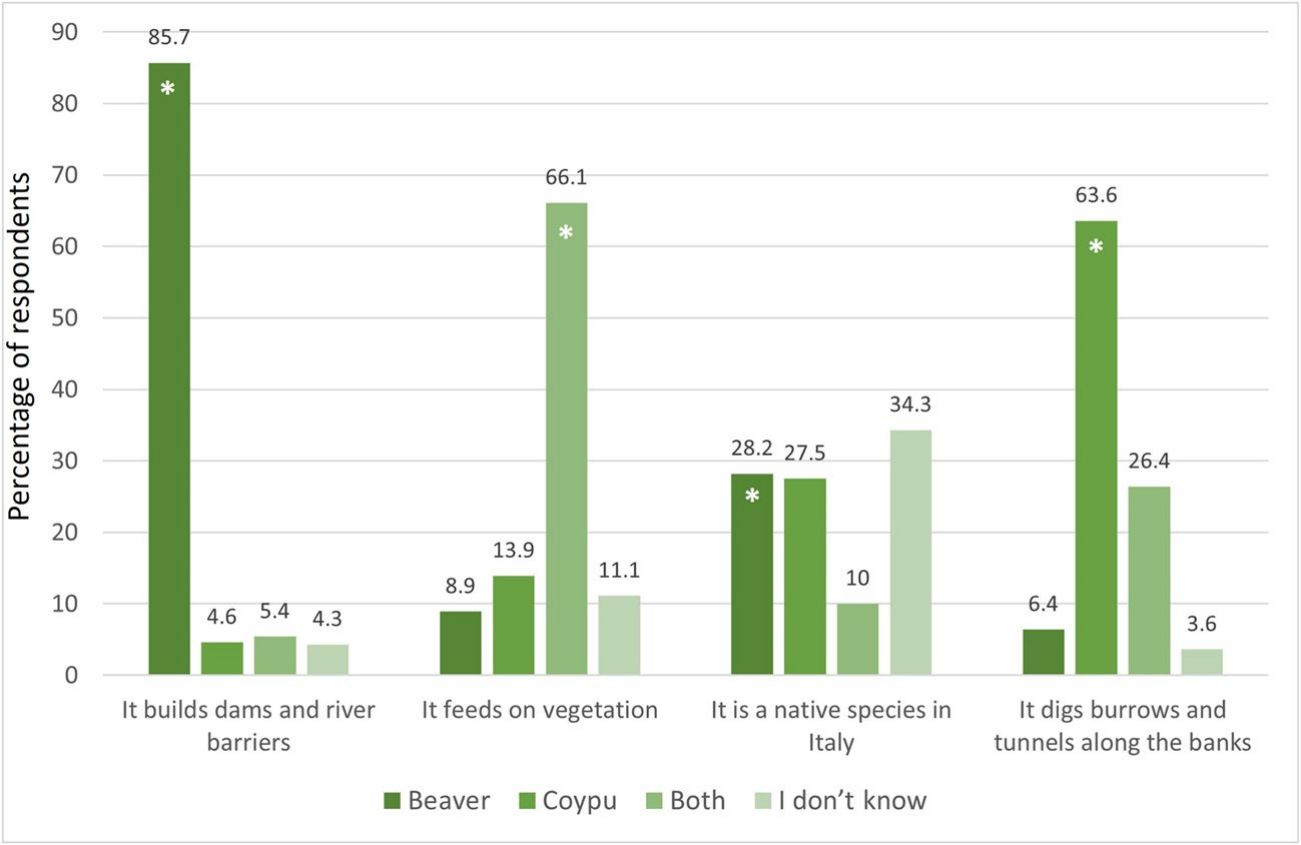
Frequency distribution (% of respondents) of answers to four statements related to coypu and/or beaver. Asterisks show correct answers

About 99.3% of respondents had heard of coypu before this survey while only 0.7% had never heard of it. According to the respondent answers, the main sources of information about coypu were the following: talking to friends/acquaintances (45.5%); traditional media, such as newspapers/radio/television (18.7%); internet/blogs (12.3%); technical-scientific articles (10.1%); conferences/meetings (4.4%). The remaining 9.0% of respondents indicated other sources of information. The high level of awareness regarding coypus is underscored by the fact that 93.9% of respondents could distinguish this species from the beaver. Furthermore, the findings also revealed a satisfactory level of understanding regarding the specific characteristics that differentiate the two species, as illustrated in Fig. 1.

Analysing the level of knowledge based on the socio-demographic characteristics, the results showed some interesting differences. Considering the gender of the respondents, a higher percentage of males affirmed that the beaver is a native species (33.7%); conversely, a greater number of females considered the coypu as native in Italy (31.8%). Considering the age, a high percentage of respondents over 64 stated that only coypu eats vegetation (38.9%), while the respondents of other age classes claimed that both the beaver and the coypu feed on riparian vegetation. For the other three socio-demographic characteristics considered in the survey – level of education, occupation, city of residence – no difference in the level of knowledge was found.

The Chi-square (χ^2^) test showed statistically significant differences among age classes for the statements “It feeds on vegetation” (*p* = 0.021) and “It digs burrow and tunnels along the banks” (*p* = 0.007).

### Perceptions and emotions towards coypu

The results showed that 38.7% of respondents were pleased to meet the coypu in the Serravalle urban park, while 34.1% were indifferent to the presence of this species and 27.2% were disturbed. Observing the data by socio-demographic characteristics, the results evidenced that a higher percentage of females (42.0%) appreciated the presence of the coypu compared to males (32.7%), the latter being mostly indifferent to its presence (41.3%). Interestingly, residents in the Empoli municipality were bothered by the presence of coypu (28.7%) more than non-residents (24.8%), as-well-as younger versus older respondents. In particular, respondents over 44 years of age were more pleased than other age groups with the presence of coypu (43.9% of respondents between 45–54 years old, 44.1% between 55–64 years old, and 44.4% over 64 years old), while the respondents under 25 were mainly indifferent (75.0%). In addition, the respondents with a higher level of education (university or post-university degree) perceived the coypu in the Serravalle urban park more negatively (36.8% are disturbed by the presence of this species, while 33.8% are pleased by its presence) compared to the respondents with a lower level of education (52.2% of respondents with an elementary/technical school degree and 41.1% with a high school degree respectively were pleased by the coypu presence). The Chi-square (χ^2^) tests showed statistically significant differences about the perception towards coypu only among age classes (*p* = 0.040) and levels of education (*p* = 0.010).

**Fig. 2:**
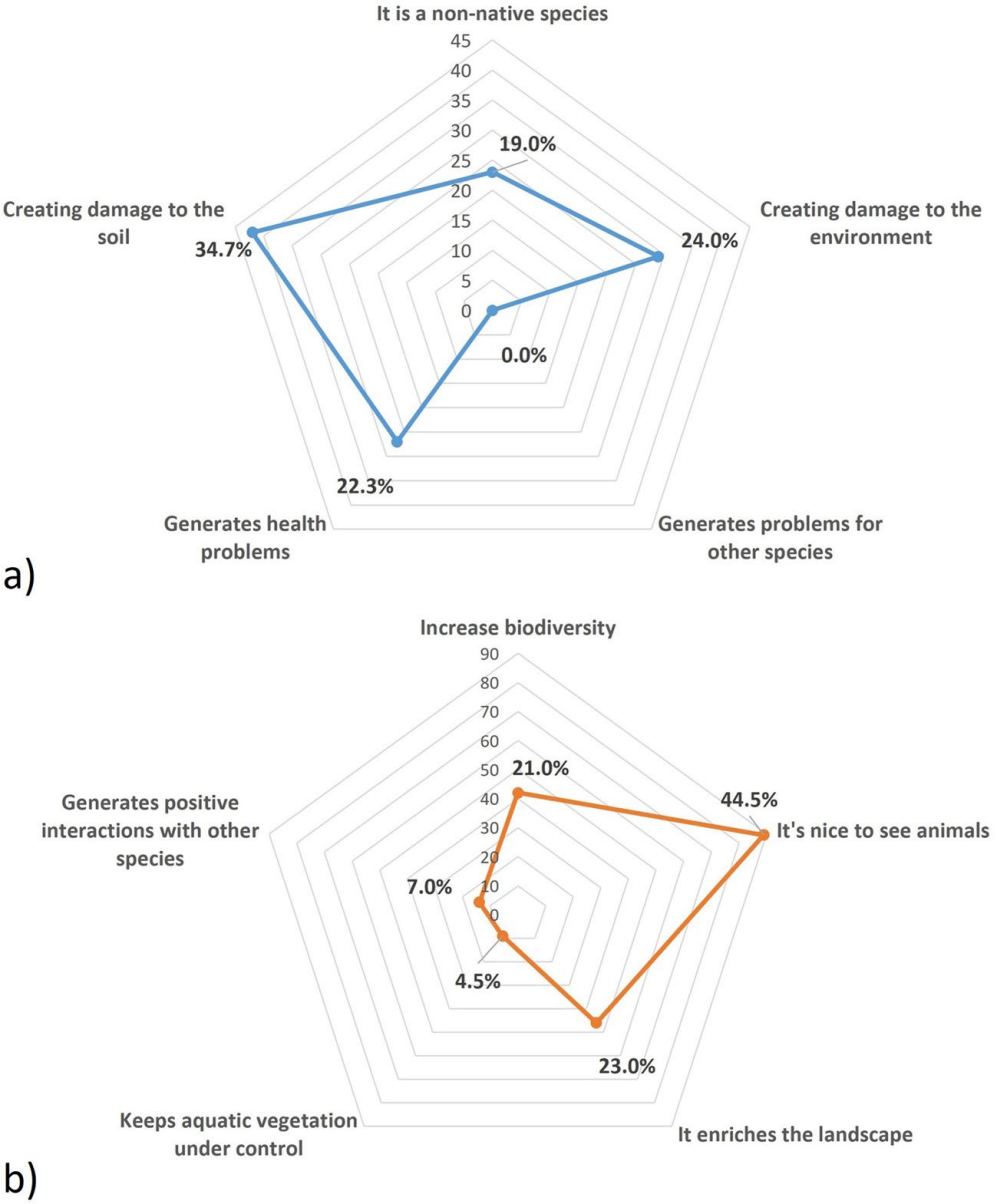
Percentage of respondents for (**a**) negative and (**b**) positive perceptions towards the coypu in Serravalle urban park, Italy

Concerning the reasons for a negative perception of coypu (Fig. 2), respondents highlighted three main factors: “Creating damage to the soil” (34.7% of respondents); “Creating damage to the environment” (24.0%); and “Generates health problems” (22.3%). Conversely, the positive perception towards coypu in the Serravalle urban park was mainly related to the statement “It’s nice to see animals” (44.5%), followed by “It enriches the landscape” (23.0%) and “Increase biodiversity” (21.0%).

The respondents’ emotions towards coypu were assigned using a seven-point Likert scale, from 0 (no emotion) to 6 (very strong emotion) and with the support of an image of this species. The results showed that the main emotion associated with the image of coypu is “joy” (mean value of 3.2 ± 2.1), followed by “love” (2.8 ± 2.0) and “disgust” (2.6 ± 2.1). Conversely, the feeling of “guilt” is the one with the lowest value (1.6 ± 1.4). Observing the data by socio-demographic characteristics (Table 2), it is interesting to emphasize that females assigned higher average values to both positive (i.e., “joy” and “love”) and negative feelings (i.e., “fear” and “disgust”) compared to males. The non-parametric Mann-Whitney test showed statistically significant differences between males and females for two feelings: “love” (*p* = 0.038) and “fear” (*p* = 0.047). About the age, the non-parametric Kruskal-Wallis test showed statistical significant differences only for one feeling: “compassion” (*p* = 0.007). Specifically, this sentiment is more pronounced among respondents aged over 64 years compared to respondents in other age brackets. Regarding the level of education, the results highlighted that respondents with a higher level of education (university or post-university degree) emphasize more the negative emotions (i.e., “fear” and “disgust”), while those with a lower level of education (elementary or technical school degree) assign higher values to positive feelings (i.e., “joy” and “love”). The Kruskal-Wallis non-parametric test confirmed statistically significant differences between respondents with different levels of education only for the two positive emotions: “joy” (*p* = 0.015) and “love” (*p* = 0.044). Conversely, the city of residence and the occupation are two variables that do not explain the differences between groups of respondents as highlighted by the Mann-Whitney and Kruskal-Wallis non-parametric tests.

**Table 2:** Respondents’ emotions towards the coypu using a seven-point Likert scale format (mean ± SD)

Emotion	Joy	Fear	Guilt	Disgust	Love	Compassion
*Gender*						
Female	**3.3 ± 2.1**	**2.2 ± 1.7**	1.5 ± 1.2	**2.7 ± 2.1**	**2.9 ± 2.1**	**2.7 ± 1.9**
Male	3.0 ± 1.9	1.7 ± 1.2	**1.9 ± 1.7**	2.5 ± 2.1	2.4 ± 1.8	2.3 ± 1.7
*Age*						
18–25 years old	2.6 ± 1.4	1.8 ± 0.8	1.3 ± 0.6	2.6 ± 1.6	2.2 ± 1.1	2.6 ± 1.3
25–34 years old	3.2 ± 2.2	**2.2 ± 1.7**	1.6 ± 1.4	2.8 ± 2.1	2.7 ± 2.1	2.6 ± 1.8
35–44 years old	3.1 ± 2.1	2.0 ± 1.7	1.7 ± 1.6	2.7 ± 2.2	2.7 ± 1.9	2.6 ± 1.9
45–54 years old	3.3 ± 2.2	2.0 ± 1.6	1.7 ± 1.5	2.5 ± 2.2	3.1 ± 2.2	2.9 ± 1.9
55–64 years old	3.1 ± 2.0	1.8 ± 1.3	1.4 ± 1.1	2.3 ± 2.0	2.6 ± 2.0	1.8 ± 1.4
> 64 years old	**3.6 ± 2.4**	1.9 ± 1.8	**2.3 ± 1.8**	**2.9 ± 2.4**	**3.4 ± 2.4**	**3.7 ± 2.8**
*Level of education*						
Elementary	**3.8 ± 2.0**	1.7 ± 1.4	**2.0 ± 1.7**	2.3 ± 2.1	**3.3 ± 2.0**	**2.8 ± 2.2**
High school degree	3.4 ± 2.2	1.9 ± 1.3	1.4 ± 1.2	2.4 ± 1.9	3.0 ± 2.1	2.7 ± 1.9
University/post-university degree	2.8 ± 1.9	**2.1 ± 1.8**	1.7 ± 1.6	**2.9 ± 2.2**	2.5 ± 1.9	2.5 ± 1.8
*Occupation*						
Public and private employees	3.1 ± 2.1	1.9 ± 1.6	1.6 ± 1.4	2.5 ± 2.1	2.8 ± 2.0	2.5 ± 1.8
Unemployed	3.2 ± 2.5	**2.2 ± 2.0**	1.5 ± 1.5	2.7 ± 2.3	2.9 ± 2.5	2.4 ± 1.7
Students	3.1 ± 1.7	1.7 ± 1.0	1.6 ± 1.3	2.8 ± 2.1	2.6 ± 1.7	2.8 ± 1.4
Retired	**3.3 ± 2.1**	2.2 ± 1.8	**2.3 ± 1.8**	**3.1 ± 2.4**	**3.1 ± 2.1**	**3.5 ± 2.7**
*City of residence*						
Inside Empoli municipality	3.0 ± 2.0	1.9 ± 1.6	**1.7 ± 1.5**	**2.7 ± 2.2**	2.7 ± 1.9	**2.6 ± 1.9**
Outside Empoli municipality	**3.3 ± 2.1**	**1.9 ± 1.6**	1.5 ± 1.3	2.4 ± 1.9	**2.9 ± 2.1**	2.6 ± 1.9

In bold the highest average values per group of respondents

### Preferences towards coypu management strategies

The results about the management of the coypu in the Serravalle urban park showed that most of respondents are against the removal of coypu (44.6%), while 13.2% are very favourable, 15.7% are favourable, and the remaining 26.4% have no opinion on the matter. Observing the preferred management strategy within the two groups (pros *vs*. cons), the results showed that among the removal strategies, the preferred one is the sterilization, with a mean value of 3.96 (SD = 2.05), compared to the strategy of capture and population control (2.9 ± 1.7). Among the conservation strategies (which are illegal, given the importance of coypu removal following the EU Regulation 1143/2014), the preferred one is the demographic monitoring through capture and marking (3.6 ± 2.0), followed by the capture and move to a controlled and circumscribed context (2.7 ± 1.7). Examining the data by socio-demographic characteristics of respondents (Table 3), the results underscored several intriguing disparities, which can be summarised as follows: males assigned a higher value to the “capture and population control” strategy compared to females (Mann-Whitney test: *p* = 0.009); respondents with a higher level of education assigned a higher value to the “sterilization” strategy and a lower value to the “free evolution without monitoring” strategy compared to respondents with other levels of education (Kruskal-Wallis test: *p* = 0.029 and *p* = 0.039 respectively); the residents in the Empoli municipality emphasized the importance of the “capture and population control” strategy more than non-residents (Mann-Whitney test: *p* = 0.036). Regarding the age, the results showed that older emphasized more the “capture and population control” strategy among the removal strategies compared to the other age classes, while younger preferred the “capture and move to another context” strategy among the conservation strategies. The non-parametric Kruskal-Wallis test did not highlight statistical significant differences among age classes for all management strategies. Similarly, no interesting statistical difference was found considering the occupation of the respondents.

**Table 3:** Respondents’ preferences towards management strategies of the coypu in the Serravalle urban park, Italy, using a Likert scale format (mean ± SD)

Strategy	Removal strategies (N = 172 respondents)		Conservation strategies (N = 109 respondents)		
	Capture and population control	Sterilization	Free evolution without monitoring	Capture and move to another context	Demographic monitoring through capture and marking
*Gender*					
Female	2.5 ± 1.3	**4.1 ± 2.0**	2.2 ± 1.4	2.7 ± 1.7	**3.7 ± 2.1**
Male	3.5 ± 2.0	**3.7 ± 2.1**	2.3 ± 1.4	2.7 ± 1.6	**3.2 ± 1.8**
*Age*					
18–25 years old	1.0 ± 0.3	**4.3 ± 1.6**	1.4 ± 0.8	**3.1 ± 1.7**	2.8 ± 1.6
25–34 years old	2.7 ± 1.6	**4.2 ± 2.2**	1.9 ± 1.3	2.1 ± 1.5	**3.6 ± 2.1**
35–44 years old	2.7 ± 1.7	**4.1 ± 2.1**	2.5 ± 1.6	3.0 ± 1.8	**3.1 ± 1.9**
45–54 years old	3.4 ± 1.8	**4.1 ± 2.0**	2.1 ± 1.3	2.5 ± 1.5	**3.6 ± 2.0**
55–64 years old	3.1 ± 1.6	**3.7 ± 1.9**	2.6 ± 1.5	3.2 ± 1.8	**4.0 ± 2.1**
> 64 years old	**4.2 ± 2.1**	2.7 ± 1.8	2.7 ± 1.8	2.6 ± 1.7	**3.8 ± 2.2**
*Level of education*					
Elementary/technical school degree	2.8 ± 1.6	2.8 ± 1.6	3.0 ± 2.0	2.3 ± 1.5	**3.9 ± 2.2**
High school degree	2.7 ± 1.4	**3.6 ± 1.9**	2.4 ± 1.6	2.7 ± 1.8	**3.4 ± 2.1**
University/post-university degree	3.2 ± 1.8	**4.3 ± 2.2**	1.7 ± 0.9	2.8 ± 1.5	**3.6 ± 1.9**
*Occupation*					
Public and private employees	2.9 ± 1.6	**4.1 ± 2.0**	2.2 ± 1.4	2.7 ± 1.7	**3.6 ± 2.0**
Unemployed	2.9 ± 1.8	**3.7 ± 2.1**	**3.4 ± 1.9**	2.4 ± 1.6	2.8 ± 1.7
Students	2.4 ± 1.3	**4.2 ± 2.1**	1.8 ± 1.2	2.5 ± 1.5	**3.1 ± 1.8**
Retired	**4.3 ± 2.1**	2.5 ± 1.7	2.4 ± 1.7	2.8 ± 1.8	**4.2 ± 2.3**
*City of residence*					
Empoli municipality	3.3 ± 1.8	**3.8 ± 2.0**	2.3 ± 1.4	2.7 ± 1.6	**3.7 ± 2.0**
Outside Empoli municipality	2.4 ± 1.4	**4.1 ± 2.0**	2.3 ± 1.4	2.7 ± 1.7	**3.3 ± 2.0**

In bold the highest average values per group of respondents

### Correlations between emotions and acceptance of management strategies

The results of the Spearman correlation test (*α* = 0.05) are shown in Table 4. These results highlighted that a higher level of knowledge is positively correlated with management aimed at removing coypu from the Serravalle urban park (*p* = 0.025) and negatively correlated with the emotion “love” (*p* = 0.046), while the negative perception of respondents towards coypu is positively correlated with the management aimed at removing the coypu (*p* < 0.0001) and with some negative emotions (“fear” *p* < 0.0001; “guilt” *p* = 0.001; “disgust” *p* < 0.0001). Besides, it is interesting to point out that management aimed at removing coypu is positively correlated with the negative emotions towards this species (“fear”, “guilt”, and “disgust” with *p* < 0.0001) and negatively correlated with the positive emotions (“joy” and “love” with *p* < 0.0001). As expected, the positive emotions towards the coypu are negatively correlated with the negative emotions (joy-fear; joy-disgust; joy-compassion; fear-guilt with *p* < 0.0001; fear-love with *p* = 0.004) and positively correlated with the other positive emotions (joy-love with *p* < 0.0001).

**Table 4:** Matrix of the Spearman correlation test (*α* = 0.05) among respondents’ knowledge, perception, emotions and management strategies

	Knowledge	Perception	Management	Emotions					
				Joy	Fear	Guilt	Disgust	Love	Compassion
Knowledge	-	0.114	0.134*	-0.040	-0.080	0.090	-0.025	-0.119*	-0.059
Perception	0.114	-	0.704***	-0.713***	0.391***	0.197**	0.662***	-0.578***	-0.045
Management	0.134*	0.704***	-	-0.588***	0.313***	0.251***	0.543***	-0.488***	-0.071
Joy	-0.040	-0.713***	-0.588***	-	-0.241***	-0.020	-0.545***	0.796***	**0.292**
Fear	-0.080	0.391***	0.313***	-0.241***	-	0.293***	0.583***	-0.174**	0.210***
Guilt	0.090	0.197**	0.251***	-0.020	0.293***	-	0.275***	0.104	0.400***
Disgust	-0.025	0.662***	0.543***	-0.545***	0.583***	0.275***	-	-0.430***	0.034
Love	-0.119*	-0.578***	-0.488***	0.796***	-0.174**	0.104	-0.430***	-	0.412***
Compassion	-0.059	-0.045	-0.071	**0.292**	0.210***	0.400***	0.034	0.412***	-

^*^p < 0.05, **p < 0.001, *** *p* < 0.0001

**Fig. 3:**
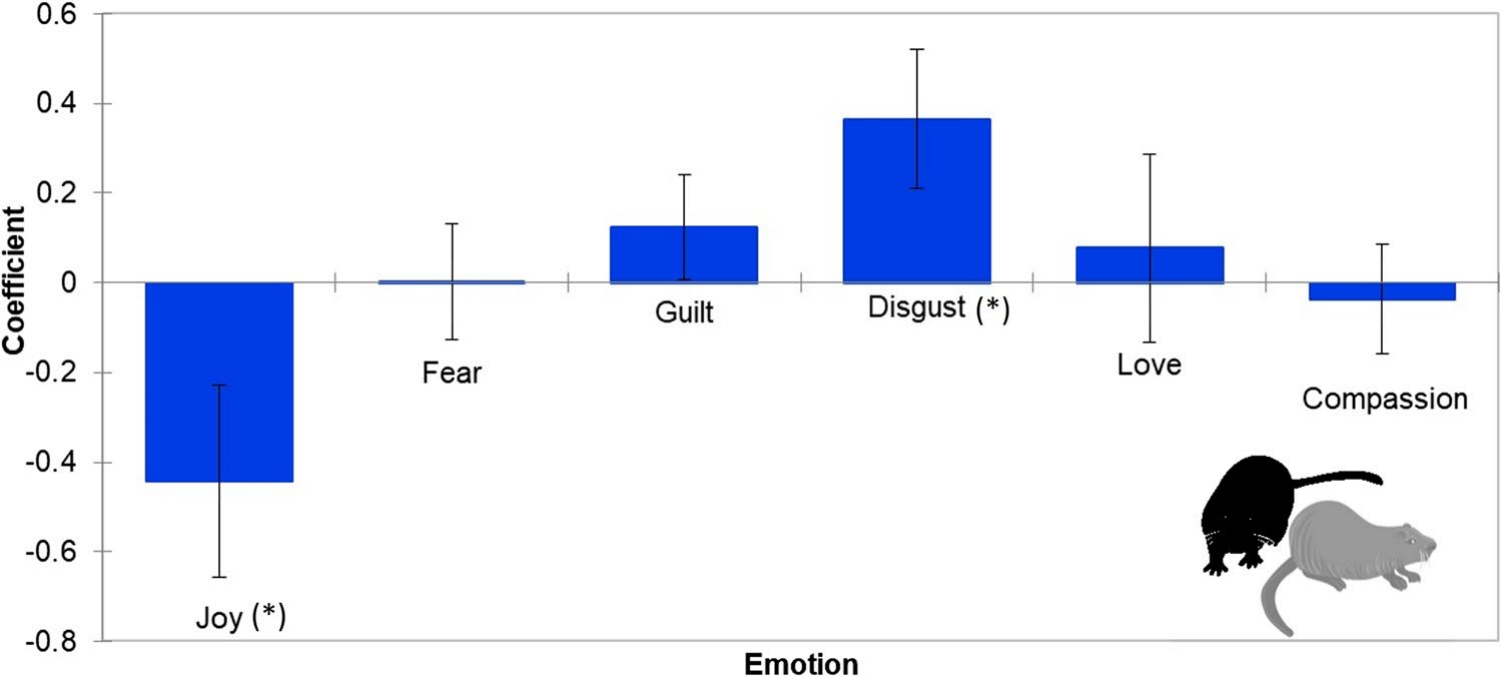
Boxplots of linear model regression for each tested emotion towards coypus in Serravalle urban park. Bars show the 95% confidence intervals; (*), *p* < 0.05

The results of the linear regression model showed that positive emotions have negative coefficients with management aimed at removing coypus, whereas negative emotions have positive coefficients, although only two emotions (namely, “joy” and “disgust”) are significant (*p* < 0.0001; Fig. 3).

## Discussion

The results of the present survey showed a high awareness on coypu, as nearly all respondents had heard about it. In particular, the vast majority of respondents were able to distinguish between coypu and beaver. In addition, interacting with friends or acquaintances emerged as the primary source of information about coypu. In general, younger respondents were more indifferent to coypu presence, whereas older respondents were usually more likely to appreciate it. Furthermore, respondents with higher education were more likely to view coypu negatively, and to agree with strong management actions (i.e., removal strategies). Conversely, older people showed a misleading perception, favouring conservation strategies (illegal for coypus in invaded European countries). This result may indicate that the increased available information and education about invasive species could help with attitudes towards these animals and contribute to people's perceptions of invasive species management (García-Llorente et al. 2008). In recent years, several European Life projects (e.g., Life CSMon, Life ASAP) have placed a strong emphasis on the communication component on biological invasions, which have also been included in a number of university curricula. This may have contributed to increased awareness, particularly among young generations (who generally have easy access to streaming information and social media) and citizens with higher levels of education, providing a strong support to the importance of an effective communication for wildlife management (Haley et al. 2023). Our study is the first in the scientific literature dealing with the social perception of coypu, one of the most widespread alien species worldwide, in an urban area, where contacts with humans are frequent. As argued by different studies (Estévez et al. 2015; Kapitza et al. 2019), it is essential to consider the social perception on wildlife management, as this can strongly influence the success of biodiversity and conservation programmes or any related action (e.g., containment actions of invasive species).

We are aware that our questionnaire survey includes a remarkable geographic limitation (i.e., a sample bias), as the survey was only distributed to visitors of the Serravalle urban park. This means the responses only represent the opinions of people who frequent the park, and not necessarily the broad human population. However, the Serravalle park offers a unique occasion to test for human perception towards management strategies against an invasive alien species, occurring with an isolated population in a highly-visited urban park. Moreover, people who have strong opinions about coypus, either positive or negative, may be more likely to take the survey, whereas those having mild opinion may have rejected it, although we recorded less than 5% rejections. Furthermore, closed-ended questions with predetermined answer choices may restrict respondent ability to express nuanced opinions or provide detailed explanations for their feelings towards coypus, despite providing a better dataset for statistical analysis.

In Italy, about 16.8% of the 125 mammal species are alien species (Loy et al. 2019), including seven species of European conservation concern (the coypu, the northern raccoon *Procyon lotor,* the raccoon dog *Nyctereutes procyonoides*, the grey squirrel, the Pallas’s squirrel *Callosciurus erythraeus,* the Finlayson’s squirrel *Callosciurus finlaysonii* and the Siberian chipmunk *Eutamias sibiricus*), for which it is necessary to activate strategies for numerical control or eradication (cfr. Anderson et al. 2022). Consequently, it is essential to develop strategies tailored to the perceptions in various social contexts, ensuring their shared acceptance by public opinion (Walther et al. 2011).

Regarding the emotional dimension of social perception, this study showed that the main emotions associated with images depicting coypu are positive, such as “joy” and “love”, while only a smaller number of respondents assigned negative emotions, such as “disgust” and “fear” to this species. These results are in line with international literature about public perception towards animal species in general and animal alien species in particular. Prokop and Randler (2018) highlighted that the historical evolution of human emotions is strictly related to the physiological and psychological responses for survival. Therefore, “fear” and “disgust” are usually the two most common human emotions towards wildlife, because they are a defence mechanism against dangerous animals (Dalgleish 2004). Conversely, wildlife species perceived as harmless (e.g., birds or small herbivore mammals), or other generally dangerous species possessing charismatic traits or not posing a significant threat to modern humans (e.g., many large carnivores, but also species represented by cartoon characters), tend to elicit positive emotions (Powell and Bullock 2014; Dydynski and Mäekivi 2021; Cerri et al. 2020; Viviano et al. 2023). As animals become a risk to human health, social perception changes to highly negative, and most forms of eradication, including lethal forms, are accepted (Garba et al. 2014; Liordos et al. 2017; Panti-May et al. 2017; Anderson et al. 2022).

About coypu, Adriani et al. (2011) highlighted that there is little clarity about the legal status of coypu and its classification as an invasive species, as the present study confirmed, and that this knowledge among general public is strongly related to the presence of information campaigns and containment programs on the territory. Therefore, their suggestion is to combine the implementation of management strategies with a greater dissemination of the main information regarding coypu among the civilian population. Therefore, the purpose of the present study appears in line with these indications. In fact, the conclusive findings of this study will be shared with both the public administration, to outline the most useful management strategies for coypu, and with the population of the municipality of Empoli. This purpose aims to disseminate knowledge about this species and, consequently, promote the most appropriate behaviour among citizens.

Regarding coypu management strategies, the results of this study highlighted a preference for sterilisation over population control. Liordos et al. (2017) found that when crop damage is evident, the lethal control of coypu is preferred by the stakeholder categories of farmers and farmers-hunters, whereas the general public is predominantly opposed to lethal control. Sterilisation of populations of invasive alien species would be effective only for small and isolated populations, located in areas with scarce reinvasion probability (e.g., grey squirrels *Sciurus carolinensis* in Liguria, NW Italy: Scapin et al. 2019). Similarly, the confinement of invasive alien species in captivity may be considered only when alien populations consist of a limited number of individuals and appropriate fenced structures are present in their surrounding (e.g., feral llamas *Lama glama* in Central Italy: Gargioni et al. 2021).

Regardless the strategies adopted by decision-makers for coypu population control, it is crucial to bear in mind that management actions not accepted by the public tend to be ineffective and costly in terms of both economic and human resources (cf. Viviano et al. 2023).

## Concluding remarks

The present study brings a deeper knowledge in the field of the social perception of the presence of coypu in the urban environment. For decision makers it is essential to effectively address the social dimension linked to the management of invasive species, particularly when the general public and animal right groups may hinder removal campaigns (cfr. Cerri et al. 2020). Accordingly, less than one-third respondents would agree with any population control, mostly for respondents over 45 years-old and with a low education level, suggesting that further information is needed to clarify the importance of removing alien species to preserve native biodiversity. Particularly, any removal campaign (eradication or numerical control) should be preceded by encounters between public administrations, wildlife managers and the general public, to explain reasons for management actions.

The coypu population of the Serravalle urban park represent an almost isolated nucleus of this species, with poor connections with other populations. Therefore, a local eradication would be possible (see Bertolino et al. 2005), but it would require an improved public knowledge about coypu invasive status to inform the local population about risks related to biological invasions. Sharing our results with both public administration and the local population will be crucial for developing an effective co-management plan. The methodology employed lends itself to potential replication in similar local-scale contexts. Management strategies should consider the social context and public preferences, favoring non-lethal methods like sterilization when feasible. By understanding public perception and collaborating with stakeholders, successful coypu management plans can be implemented, ensuring both biodiversity conservation and public acceptance.

## Fundings

EM was funded by the National Recovery and Resilience Plan (NRRP), Mission 4 Component 2 Investment 1.4—Call for tender No. 3138 of 16 December 2021, rectified by Decree n.3175 of 18 December 2021 of Italian Ministry of University and Research funded by the European Union – NextGenerationEU; Project code CN_00000033, Concession Decree No. 1034 of 17 June 2022 adopted by the Italian Ministry of University and Research, CUP B83C22002930006, Project title “National Biodiversity Future Center—NBFC”.

## Supplementary information

Below is the link to the electronic supplementary material.

Supplementary file1 (DOCX 378 KB)

## Data Availability

Data used in this study are available in the Electronic Supplementary Materials.
