# Supplementary material for: Public perception and acceptance of coypu Myocastor coypus removal in urban areas: influences of age and education
Source: Naturwissenschaften. 2024 Aug 2;111(5):42. doi: 10.1007/s00114-024-01928-2 (PMC11297126; doi:10.1007/s00114-024-01928-2)
Supplement: Supplementary file 1 — Supplementary file1 (DOCX 378 KB) [file 114_2024_1928_MOESM1_ESM.docx]

**Public perception of the presence of coypu *Myocastor coypus* in urban environments: the case study of an urban park in Central Italy**

Viviano Andrea, De Meo Isabella, Mori Emiliano, Sergiacomi Carlotta, Paletto Alessandro

# ANNEX 1


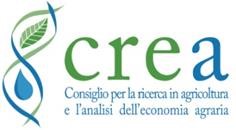

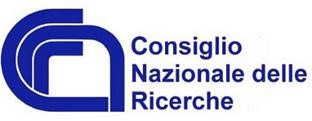


**QUESTIONNAIRE FOR VISITORS TO SERRAVALLE PARK**

The questionnaire has been realized in the framework of the National Recovery and Resilience Plan (PNRR)- National Biodiversity Future Center. The project aims at investigating different management actions for the fauna of suburban areas with high anthropization. This survey is not of a commercial or advertising nature but purely scientific; the data collected will be processed and disclosed only in aggregate form, in accordance with

Legislative Italian Decree 196/2003.

Thank you for the collaboration.

## SECTION 1 - KNOWLEDGE REGARDING THE COYPU AND ITS PRESENCE IN SERRAVALLE PARK

Q1.1. The image below represents two different species of rodents. Can you identify which of the two is a coypu?


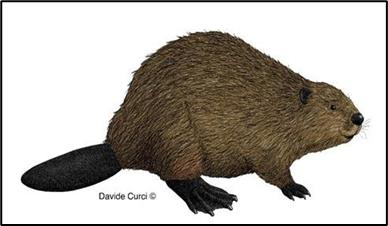





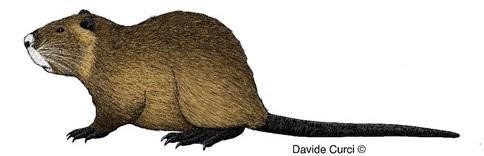


 I don’t know

Q1.2. In your opinion, do the following statements refer to coypu and/or beaver??

|  | Coypu | Beaver | Both | I don't know |
| --- | --- | --- | --- | --- |
| a - Builds dams and river barrages |  |  |  |  |
| b - It feeds on vegetation |  |  |  |  |
| c - Is a native species in Italy |  |  |  |  |
| d - Digs burrows and tunnels along riverbanks |  |  |  |  |

Q1.3. Had you ever heard / read about coypu before today?


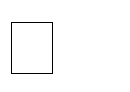
 Yes


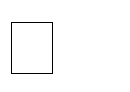
 No

Q1.4. If “YES”, where did you heard / read about coypu? (Multiple choice):


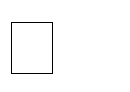
 In technical-scientific articles


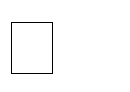
 In magazines/radio/television


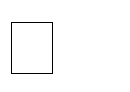
 In post on internet/blog


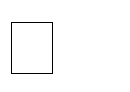
 In conferences/workshops/public meetings


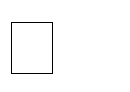
 In conferences/workshops/public meetings


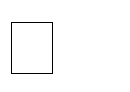
 Chatting with people I know


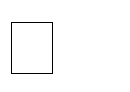
 Other: __________________________

## SECTION 2 – PERCEPTION OF THE PRESENCE OF COYPU IN SERRAVALLE PARK

Q2.1. If walking inside the Park you see a coypu like the one shown in the picture below what do you think (*single answer*)?

 I am disturbed by its presence


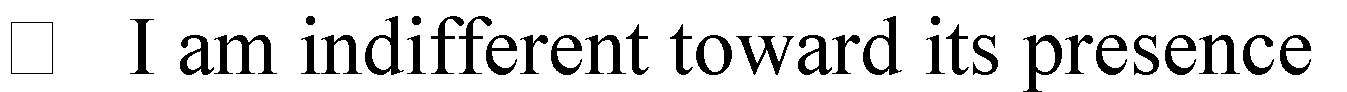

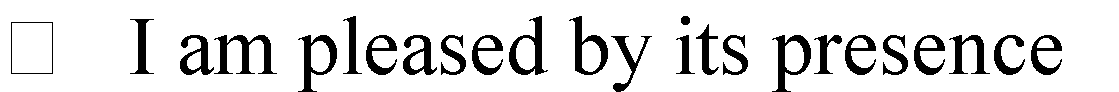


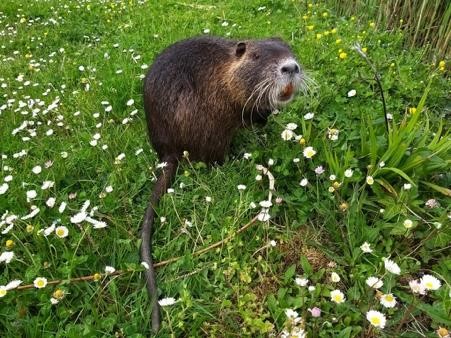


Q2.1.1 In case you answered "I am disturbed" can you give reasons for your answer (*multiple choice*)?
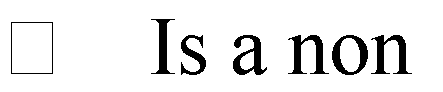
-native species in Italy


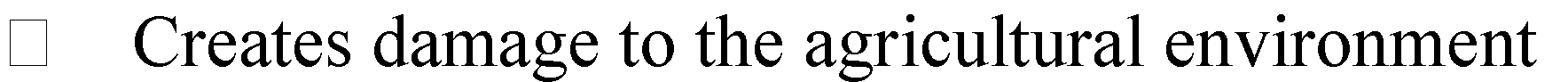


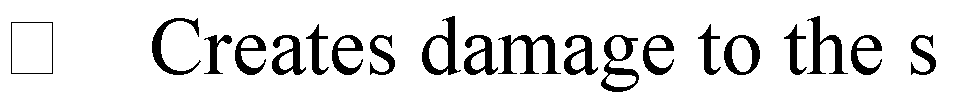
oil


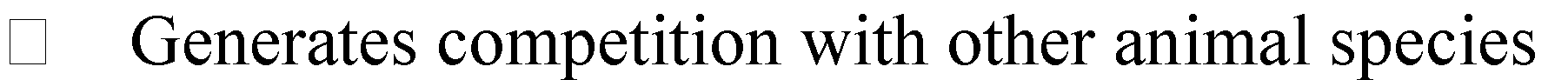

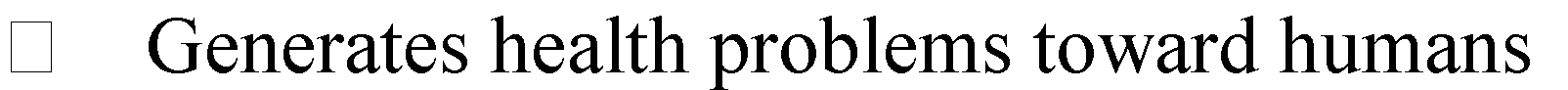

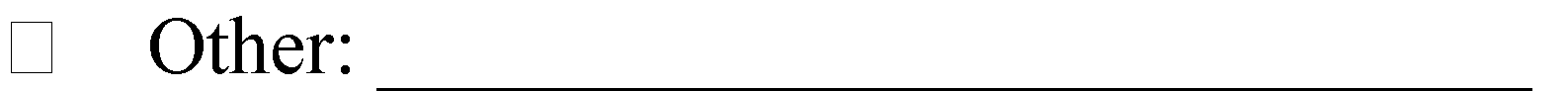


Q2.2.2 In case you answered "I am pleased" can you give reasons for your answer (*multiple choice*)?
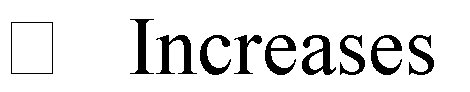
 biodiversity


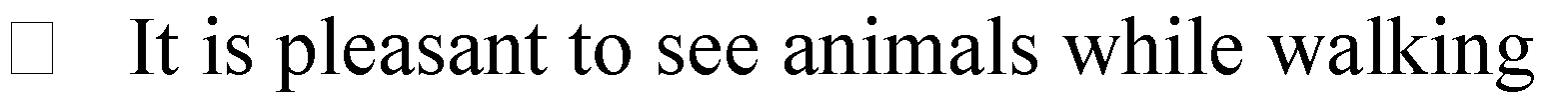

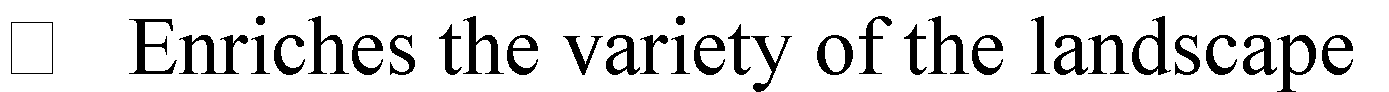

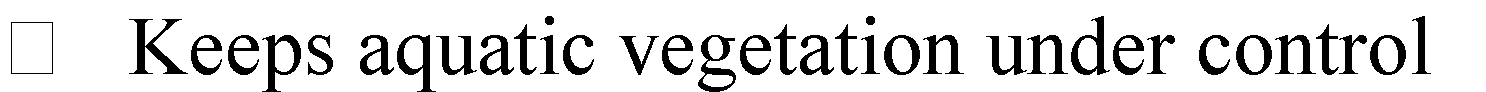

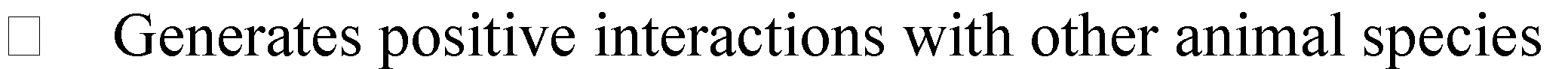


Q2.2. Looking at the above photo of a coypu, can you indicate which of the following emotions it evokes in you

(assign a value on a scale from 1 to 7 considering 1 as "no emotion" and 7 "very strong emotion")

| **Emotions** | 1 | 2 | 3 | 4 | 5 | 6 | 7 |
| --- | --- | --- | --- | --- | --- | --- | --- |
| Joy |  |  |  |  |  |  |  |
| Fear |  |  |  |  |  |  |  |
| Guilt |  |  |  |  |  |  |  |
| Repugnance/Disgust |  |  |  |  |  |  |  |
| Love |  |  |  |  |  |  |  |
| Compassion |  |  |  |  |  |  |  |

The coypu is a rodent native to South America (Brazil, Paraguay, Uruguay, Bolivia, Argentina and Chile) that has been introduced to Italy since the 1960s as a furbearing animal. It has since spread widely on its own due to frequent escapes from captivity and because of intentional releases.


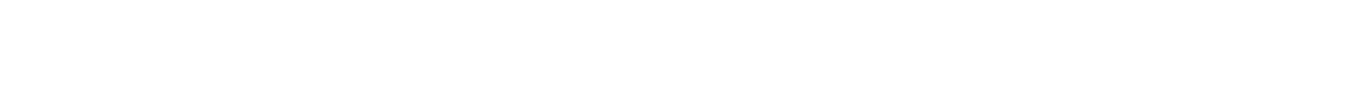


Q2.3. In light of this and of your personal thoughts, how much would you support its removal from Serravalle Park (*single answer*)?


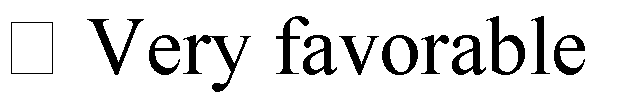

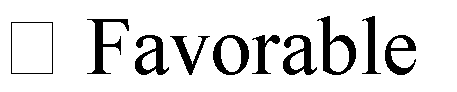

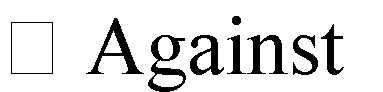

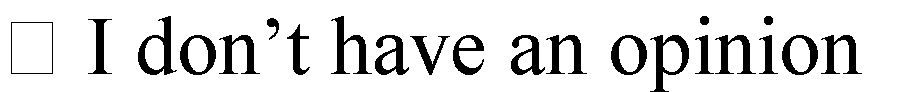


Q2.3.1 If you answered "Very favorable" or "Favorable" to the previous question, please indicate for the below methods of removal of the coypu population from an urban area your level of approval (assign a value on a scale from 1 to 5 considering 1 as total disapproval and 5 as total approval)

| **Removal strategies** | 1 | 2 | 3 | 4 | 5 |
| --- | --- | --- | --- | --- | --- |
| Direct capture and suppression |  |  |  |  |  |
| Sterilization |  |  |  |  |  |

Q2.3.1 If you answered "Against" to the previous question, please indicate for the below methods of management of the coypu population in an urban area your level of approval (assign a value on a scale from 1 to 5 considering 1 as total disapproval and 5 as total approval)

| **Conservation strategies** | 1 | 2 | 3 | 4 | 5 |
| --- | --- | --- | --- | --- | --- |
| Left to free evolution with no monitoring |  |  |  |  |  |
| Direct capture and movement to a confined and controlled environment (e.g., fenced area with pond) |  |  |  |  |  |
| Demographic monitoring through capture and tagging |  |  |  |  |  |

## SECTION 3- PERSONAL INFORMATION

Q3.1. What is your age?

 Less than 25 years old

 25-34 years old

 35-44 years old

 45-54 years old

 55-64 years old

 More than 65 years old

Q3.2. Gender

 Male  Female  Not binary

Q3.3. What is your level of education?

 Elementary school degree

 High school degree

 University degree

 Post-University degree

Q3.4. What is your actual job?

 Employed in the public sector

 Employed in the private sector

 Student

 Pensioner/Retired

 Unemployed

 Other

Q3.5. What is your place of residence?

 Empoli municipality

 Another municipality in Tuscany region
